# Light‐Annealed Piezoelectric Films on Flexible Glass

**DOI:** 10.1002/smsc.202500227

**Published:** 2025-06-20

**Authors:** Juliette Cardoletti, Longfei Song, Adrian‐Marie Philippe, Stéphanie Girod, Barbara Malič, Emmanuel Defay, Sebastjan Glinšek

**Affiliations:** ^1^ Smart Materials Unit Luxembourg Institute of Science and Technology 41 rue du Brill L‐4422 Belvaux Luxembourg; ^2^ Advanced Analysis and Support Unit Luxembourg Institute of Science and Technology 41 rue du Brill L‐4422 Belvaux Luxembourg; ^3^ Electronic Ceramics Department Jožef Stefan Institute Jamova cesta 39 1000 Ljubljana Slovenia; ^4^ Department of Physics and Materials Science University of Luxembourg 41 rue du Brill L‐4422 Belvaux Luxembourg

**Keywords:** flash lamp annealing, flexible glass, piezoelectric thin films

## Abstract

The functionalization of thin, flexible glass with piezoelectric oxides is a pathway toward transparent electromechanical devices. The crystallization of lead zirconate titanate thin films on thick, rigid glass is previously demonstrated using flash lamp annealing to selectively anneal the films, without damaging the substrates. In this work, a 2‐step process suitable for Schott AF 32 eco glass and Corning Willow glass is developed, both 100 μm thick, the latter of which is compatible with roll‐to‐roll processes. Herein, demonstration is done of: 1) the importance of heat management during flash lamp annealing; and 2) a method to orient perovskite thin films during their crystallization by flash lamp annealing. With this process, the 540 nm thick lead zirconate titanate thin films with morphotropic phase boundary composition display a relative permittivity *ε*_r_ of 330, a dielectric loss of 8.4% and a piezoelectric coefficient $e_{33\text{,f}}$ of 5.5 C m^−2^. This work demonstrates the feasibility of transparent piezoelectric films, which has a potential to opening the way to flexible and invisible devices.

## Graphical abstract

This work demonstrates the functionalization of flexible glass with piezoelectric oxides as a pathway toward transparent electromechanical devices. Using flash lamp annealing to selectively anneal the films without damaging the substrates, a process is developed for thin glass substrates, demonstrating the importance of heat management and a method to orient perovskite thin films during their crystallization by flash lamp annealing.

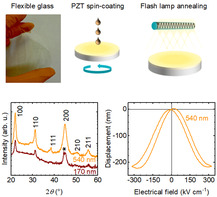

## 1 Introduction

Piezoelectric thin films on flexible substrates have attracted a strong interest in the last years for their potential applications as wearable electronic devices,^[^ ^1^ ^]^ energy harvesters^[^ ^2^ ^]^ and sensors.^[^ ^3^ ^]^ Flexible substrates offer key advantages, including the capability to conform to curved surfaces,^[^ ^4^ ^]^ greater mechanical flexibility and a reduced mass. Beyond their applications, flexible substrates are interesting for manufacturing, especially for roll‐to‐roll processes.

Combining piezoelectrics on flexible substrates and on glass substrates can pave the way to new applications, especially in the field of transparent electronics. Displays screens and functional windows, already ubiquitous, are set to become even more relevant in the coming years. The global market for smart glass, worth $7.0 billion in 2024 is predicted to reach $9.6 billion in 2030.^[^ ^5^ ^]^ The development of thin layers of transparent flexible piezoelectric devices which could be applied on any surface would be a major step to functionalize display screens and wearable devices.

For these applications, perovskite oxides stand out as excellent candidates due to their large piezoelectric coefficients. Indeed, polymers (e.g., polyvinylidene fluoride‐based materials) and nitrides (e.g., AlN) piezoelectric coefficients are less interesting for actuator applications due to their lower piezoelectric coefficients.^[^ ^6^, ^7^, ^8^ ^]^

The deposition of piezoelectric oxide thin films directly on thin, flexible glass raises several challenges: first, their high crystallization temperature, typically above 650 °C, is incompatible with the strain point (i.e., the maximum temperature before creep) of many glasses;^[^ ^9^, ^10^ ^]^ second, the amorphous nature of glasses does not allow thin‐film growth along a selected preferential crystallographic orientation, necessary to optimize the piezoelectric response.

The first challenge can be tackled by using flash lamp annealing (FLA) to selectively anneal piezoelectric thin films without bringing glass substrates above their maximum temperature. This approach was demonstrated in our previous work, where ${\text{PbZr}}_{0.53}{\text{Ti}}_{0.47}O_{3}$ (PZT) thin films were deposited by sol–gel method onto various glass substrates (fused silica glass, Schott AF 32 eco glass and soda‐lime glass).^[^ ^11^ ^]^ In FLA, sub‐millisecond light pulses of strong intensity and broad spectrum are absorbed leading to selective annealing based on the material absorptivity.^[^ ^12^ ^]^ FLA is an ultrafast annealing technique, which enables large‐scale production, unlike methods aiming to lower the processing temperature of perovskite piezoelectrics, such as combustion synthesis,^[^ ^13^ ^]^ microwave‐assisted hydrothermal reaction,^[^ ^14^ ^]^ or photochemical processing,^[^ ^15^, ^16^, ^17^ ^]^ which lead to long annealing time (tens of minutes to hours). Similar to FLA, recently developed photochemical processing methods are used after sol–gel deposition to anneal large areas, providing similar advantages, notably the flexibility to tailor the solution chemistry. However, photochemical processing methods are usually performed under controlled atmosphere^[^ ^16^, ^17^ ^]^ and remain significantly more time consuming (e.g. 30 min)^[^ ^16^, ^17^ ^]^ than FLA. Additionally, FLA ability to process large areas is a major advantage to obtain homogeneous films, contrary to laser annealing whose small spot size imposes scanning of the beam over the sample.^[^ ^18^ ^]^ Moving toward thinner (100 μm) glass, the considerable heat load induced by FLA in a very brief time span must be managed and the annealing process we developed in ref. [11] must be adapted.

The second challenge, orienting piezoelectric thin films along a selected crystallographic orientation, is generally solved by using a seed layer, for example PbTiO_3_ (PTO) for Pb(Zr,Ti)O_3_ thin films.^[^ ^19^ ^]^ With their ability to grow in a preferential orientation by themselves, seed layers allow to orient thin films, including on polycrystalline substrates.^[^ ^20^, ^21^ ^]^ However, while nucleation usually starts at the substrate–thin film interface with conventional annealing techniques like rapid thermal annealing (RTA),^[^ ^22^, ^23^ ^]^ the nucleation process during FLA has, to our knowledge, not yet been studied. Yet, understanding where nucleation occurs is crucial for the use of seed layers.

In this work, we revisit the crystallization of ${\text{PbZr}}_{0.53}{\text{Ti}}_{0.47}O_{3}$ thin films on flexible, 100 μm thick glass. Building on our previous work on 500 μm thick Schott AF 32 eco glass (AF32), we study heat management through a 1‐step process versus a 2‐step process during flash lamp annealing of PZT thin films on thin glass. The development of this 2‐step FLA process is a key advancement presented in this work, as it enables the use of very thin and flexible glass substrates. The impact of these processes on the crystallization as well as on the dielectric, ferroelectric and piezoelectric properties of PZT thin films is analyzed in Section 2.1. Afterwards, in Section 2.2, we study the use of a PTO seed layer to improve the orientation of the PZT thin films in the {100} direction and their piezoelectric response. In particular, we identify the localization of the nucleation of PZT thin films via FLA using transmission electron microscopy (TEM). We demonstrate that our process is suitable to grow thicker piezoelectric films in Section 2.3. Finally, in Section 2.4, we extend our process beyond AF32 glass, by applying it to 100 μm thick Willow glass, commercially sold as suitable for roll‐to‐roll processes.

## 2 Results and Discussion

### 2.1 Heat Management During FLA Process

**Figure 1:**
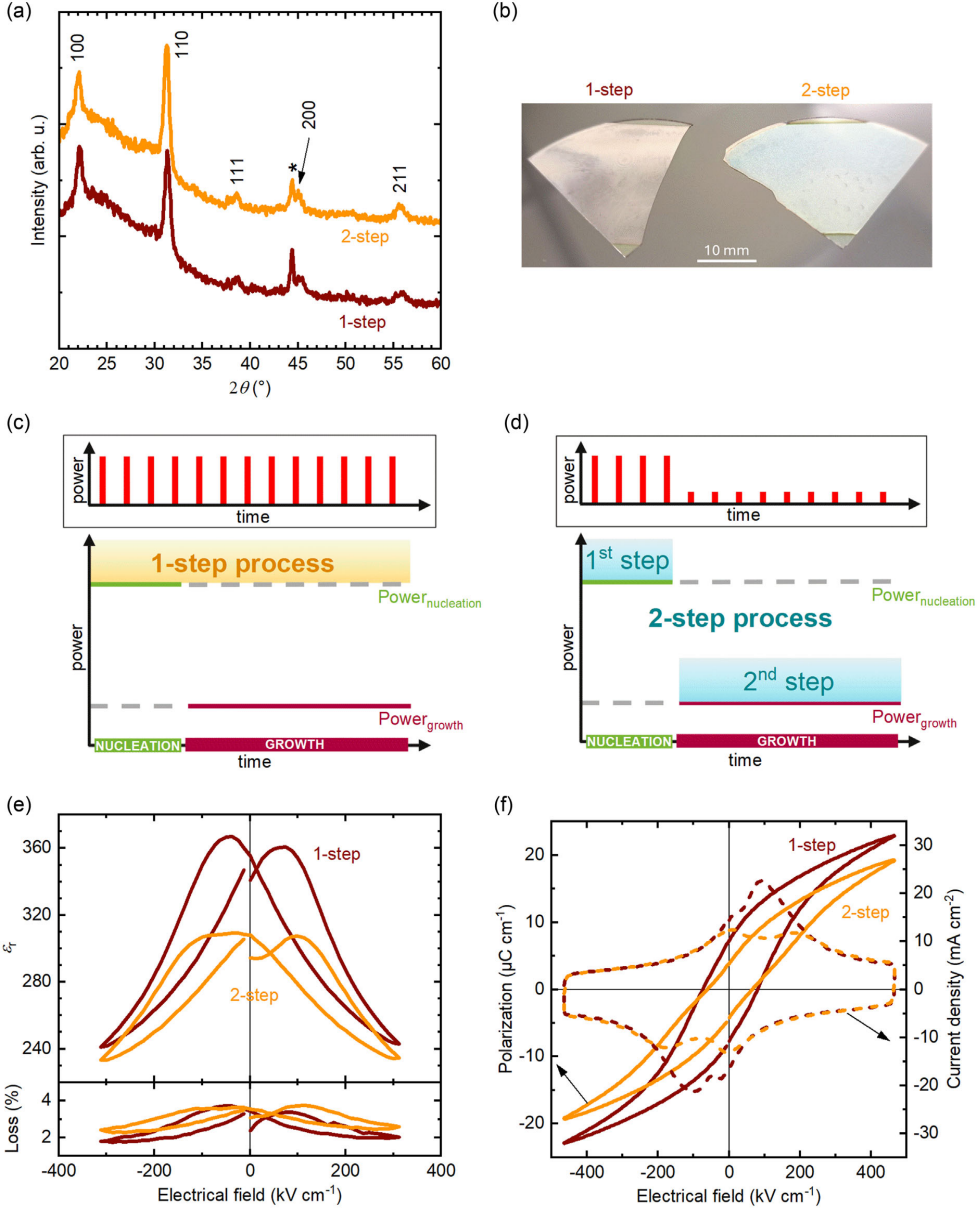
Comparison between 170 nm thick PZT films on AF32 glass annealed by FLA with 1‐step and 2‐step processes, respectively. a) θ−2θ XRD scans. The pseudo‐cubic *hkl* indexes correspond to the PDF card n°01‐070‐4264,^[^ ^37^ ^]^ while * denotes a signal from the sample holder. b) Visual aspect of the samples. The regions delimited by straight lines at the bottom and at the top of the samples correspond to the clamping positions during FLA and were not exposed to the Xe lamp. Schematic of c) the 1‐step process and d) the 2‐step process (not to scale). Power_nucleation_ and Power_growth_ are the powers for nucleation and growth, respectively. The insets represent the power of the FLA pulses over time. The number of pulses represented is arbitrary. e) Relative permittivity and dielectric loss at 1 kHz as a function of DC bias. f) Polarization (solid lines) and current density (dashed lines) versus electrical field hysteresis at 100 Hz.

In our previous work, we demonstrated that the optimal flash lamp annealing for 170 nm thick PZT films on 500 μm thick AF32 glass was a 1‐step process consisting of 50 pulses of 130 μs with an energy density per pulse of 3.0 J cm^−2^ and a repetition rate of 3.5 Hz.^[^ ^11^ ^]^ Attempts to apply the same parameters to 100 μm thick AF32 glass, that is, five times thinner, also led to the crystallization of perovskite PZT, as shown in **Figure** **1**a. However, two factors prompted us to re‐evaluate the approach: in the majority of cases, the glass broke into several pieces during annealing; and the surface of the crystallized PZT film was visually inhomogeneous, as shown in Figure 1b (1‐step sample), where two contrasted areas (white and grey) can be observed. The contrasted area has been highlighted in Figure S1, Supporting Information.

Two opposing requirements underlie the crystallization of perovskite PZT thin films by flash lamp annealing on glass: the crystallization process requires a minimum amount of energy to be delivered to the film; and the sample must be able to dissipate the excess thermal energy sufficiently fast to avoid thermal damage. The crystallization itself consists of two distinct processes: nucleation and growth of the thin film. Kwok and Desu established that their activation energies are $E_{\text{a nucleation}}$ = 441 kJ mol^−1^ and $E_{\text{a growth}}$ = 112 kJ mol^−1^, respectively.^[^ ^24^ ^]^ The first requirement for crystallizing PZT on glass is that the energy delivered to the film must be successively higher than these activation energies for nucleation and growth.

Regarding the dissipation of the thermal energy provided to the sample, it is necessary to account for the duration and, therefore, to consider the power, given in Equation (1) as:

(1) $$P=\frac{E}{t}$$

where *P* is the power density, *E* is the energy density, and *t* is the duration. To prevent thermal damage to the samples, the second requirement is that the power density per pulse must be kept at a minimum while meeting the energy requirement set above. Besides, as Kwok and Desu also showed that nucleation is brief compared to growth,^[^ ^24^ ^]^ the power density required for nucleation is larger than for growth. This is schematically represented in Figure 1c,d.

Experimentally, taking into account the energy absorption of amorphous PZT films of 28.5%,^[^ ^11^ ^]^ the 1‐step process established for 170 nm thick PZT films on 500 μm thick AF32 meets the energy requirement with an energy density per pulse larger than the activation energies for nucleation and growth of PZT. However, with an absorbed power density per pulse of 6.6 kW cm^−2^, the requirement for the dissipation of thermal energy is not met as the power density per pulse during growth is not minimized, as represented schematically in Figure 1c.

In the case of a PZT film on 100 μm thick AF32, this significant absorbed power density per pulse, combined with the successive heating‐cooling cycles associated with each FLA pulse,^[^ ^11^ ^]^ generates too much thermal strain on the sample. While thicker AF32 glass can absorb the generated heat without noticeable damage,^[^ ^11^ ^]^ this is not the case for thinner glass, which has a lower volume and therefore a lower heat capacity—the volumetric heat capacity being an intrinsic property of the glass.

To limit the amount of energy received by thin‐glass substrates, a 2‐step FLA process was engineered, similar to the method proposed for low thermal conductivity soda‐lime glass.^[^ ^11^ ^]^ As schematically described in Figure 1d, the 2‐step process consists of a first step with high power density, and of a second step with lower power density, to facilitate thermal energy dissipation. This 2‐step process allows for meeting simultaneously the activation energy and the thermal energy dissipation requirements.

**Figure 2:**
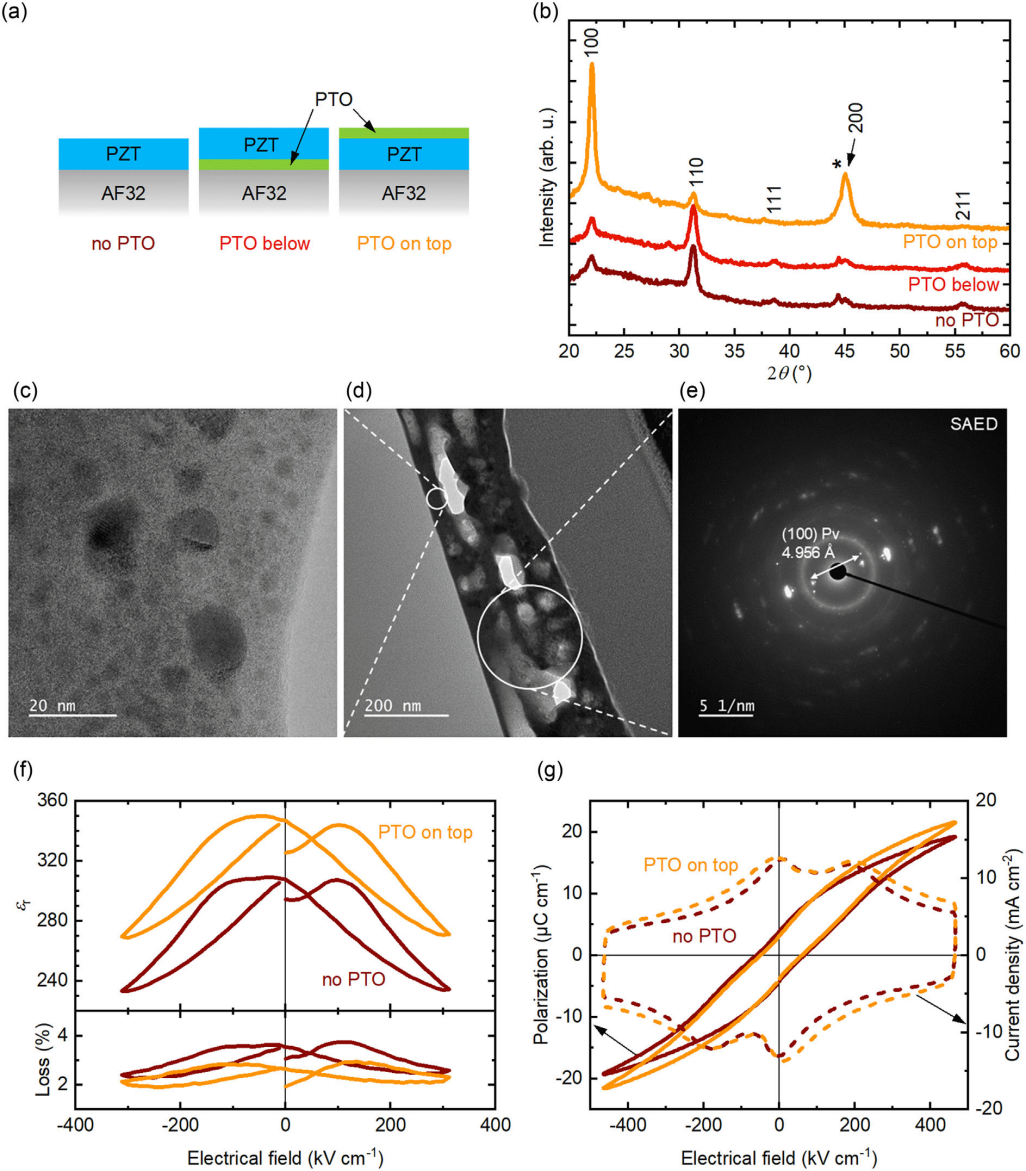
Influence of a 15 nm thick PbTiO_3_ seed layer on a 170 nm thick PZT thin film on AF32 glass. a) Schematic representation and b) θ−2θ XRD scans with various PTO seed layer configurations. The pseudo‐cubic *hkl* indexes correspond to the PDF card n°01‐070‐4264^[^ ^37^ ^]^ and * denotes a signal from the sample holder. Schematic representation not to scale. c) Zoomed region of d) TEM micrograph of a PZT thin film with a PTO layer on top. e) SAED analysis of the highlighted region. “Pv” indicates a perovskite phase. The pseudo‐cubic *hkl* indexes correspond to the PDF card n°01‐070‐4264.^[^ ^37^ ^]^ Comparison between PZT films, with and without a PTO layer on top on AF32 glass. f) Relative permittivity and dielectric loss at 1 kHz as a function of DC bias. g) Polarization (solid lines) and current density (dashed lines) versus electrical field hysteresis at 100 Hz.

Experimentally, the first step comprises 6 pulses of 170 μs with an energy density per pulse of 2.5 J cm^−2^ and a repetition rate of 0.5 Hz while the second step consists of 100 pulses of 250 μs with an energy density per pulse of 3.0 J cm^−2^ and a repetition rate of 2.0 Hz. For comparison with the 1‐step process, as summarized in **Table** **1**, the power densities per pulse absorbed by the sample are 4.2 and 3.4 kW cm^−2^ during the first and second steps, respectively. These values, providing enough power for nucleation and growth, allow for the crystallization of PZT thin films with the same results as for the 1‐step process with regard to θ−2θ X‐ray diffraction (XRD) scans, displayed in Figure 1a. On the contrary, these reductions in power density per pulse of 36% for the first step and 48% for the second step can explain why 100 μm thick AF32 glass does not break during the 2‐step FLA process. Additionally, the visually uniform crystallized surface, shown in Figure 1b, can be attributed to the reduced, and therefore more manageable, thermal load. Note that the small circular marks on the 2‐step sample are on the back of the substrate due to the shower‐head chuck used for spin‐coating. In Figure 1a, the (110) reflection (see Figure S2, Supporting Information for a zoom on this reflection) is slightly sharper for the 1‐step process than for the 2‐step process indicating slightly larger crystallite size for the 1‐step process as compared to the 2‐step process. However, the complex microstructure of the thin films (see **Figure** **2**c,d) makes quantification of the crystallite size difficult.

**Table 1:** Absorbed power density per pulse for the 1‐step and for each step of the 2‐step FLA processes to crystallize a 170 nm thick PZT film on 100 μm thick AF32 glass.

Process		Pulse duration [μs]	Energy density per pulse [J cm^−2^]	Power density per pulse [kW cm^−2^]	Absorbed power density per pulse [kW cm^−2^]
1‐step		130	3.0	23.1	6.6
2‐step	1st step	170	2.5	14.7	4.2
	2nd step	250	3.0	12.0	3.4

The dielectric properties of the PZT films crystallized via the 1‐step and the 2‐step processes are shown in Figure 1e. The relative permittivity *ε*_r_ is of 340 for the 1‐step process and 295 for 2‐step process. In both cases, the dielectric loss remains moderate, with 3.3% and 3.5% for the 1‐step and 2‐step processes, respectively.

Figure 1f shows the polarization and current density versus electrical field hysteresis loops for the 1‐step and the 2‐step FLA processes. The 1‐step process yields a remanent polarization *P*_r_ of 7.3 μC cm^−2^, a maximum polarization *P*_max_ of 22.9 μC cm^−2^ and a coercive field *E*_c_ of 80 kV cm^−1^. On the contrary, the 2‐step process has respective values of 3.7, 19.2 μC cm^−2^ and 65 kV cm^−1^ for *P*_r_, *P*_max_, and *E*_c_. The 1‐step process leads to a single switching peak at *E*_c_ with a shoulder at ≈10 kV cm^−1^. For the 2‐step process, two distinct switching peaks are observed at ≈10 and 190 kV cm^−1^. This behavior is reflected in the pinched polarization versus electrical field loop of the 2‐step process. The two switching peaks are indicative of charged defects present in the samples, which pin ferroelectric domain walls.

From both the dielectric and ferroelectric properties, it emerges that the 1‐step process is more efficient for crystallizing 170 nm thick PZT thin films on AF32 glass. However, since the 1‐step process causes the glass to break into several pieces during FLA in most cases, it cannot be considered a suitable process for PZT crystallization on 100 μm thick glass. This conclusion is further supported by the visual inhomogeneity of the films after the 1‐step process (see Figure 1b) in contrast to the 2‐step process. Therefore, the remainder of this work focuses exclusively on the 2‐step FLA process.

### 2.2 Orienting Thin Films Crystallized by FLA

The details of the crystallization process of amorphous oxide films by FLA have not been studied so far. With standard annealing methods, for example, rapid thermal annealing, nucleation typically starts at the substrate–film interface.^[^ ^22^, ^23^ ^]^ Therefore, it is possible to trigger the oriented crystallization of the film along a preferred orientation by first depositing a seed layer. For PZT thin films, PTO has been reported as a good seed layer to trigger {100}‐orientation.^[^ ^19^ ^]^

We hypothesize that, by flash lamp annealing, nucleation is triggered in the film region that first absorbs the light, that is, the top surface. To verify this hypothesis, three configurations of a 15 nm thick PTO seed layer were tested to trigger the {100}‐orientation of the PZT layer during its crystallization: 1) a PZT layer without a PTO layer (no PTO); 2) a PTO layer below the PZT layer, annealed together by FLA (PTO below); 3) a PTO layer on top of the PZT layer, annealed together by FLA (PTO on top).

A schematic representation of these three configurations is shown in Figure 2a. The FLA parameters are given in Section S3, Supporting Information.

The θ−2θ XRD scans in Figure 2b show that without a PTO layer the PZT perovskite phase is randomly oriented. This is consistent with the Lotgering factor,^[^ ^25^ ^]^ *f*, of 0.40. The configuration where a PTO layer is deposited below the PZT layer and they are annealed together by FLA leads to a randomly oriented PZT thin film with a Lotgering factor of 0.32. This value is lower than the value of 0.40 obtained without any PTO seed layer, indicating the very low quality of the PZT film with a PTO layer below. In contrast, with a PTO layer on top of the PZT layer, the thin film crystallizes with a strong {100} texture, as indicated by the Lotgering factor of 0.80.

To further confirm that, during flash lamp annealing, the nucleation of the amorphous PZT starts at its top surface, TEM analysis was performed on a 170 nm thick PZT thin film with a 15 nm thick PTO layer on top. From the overview image in Figure 2d, large pores are visible in white. The size of these pores is similar to those previously reported for PZT thin films crystallized by FLA.^[^ ^11^ ^]^ We hypothesize that their formation is due to the ultrafast annealing by FLA hindering the evacuation of gases from the thin film during annealing. The analysis of a smaller region of the sample, shown in Figure 2c, indicates the presence of spherical inclusions in an amorphous environment. These inclusions have the same chemical composition as the surrounding matrix. Reduction of both porosity and the extent of the amorphous phase could possibly be achieved through a longer pyrolysis which would promote removal of organics and increase the formation of M—O—M bonds. It should lead to a denser, more crystalline microstructure of the thin films. Furthermore, reducing the thickness of each annealed layer should contribute to a reduction in porosity. However, such modifications of the thin films processing would require significant adjustments of the 2‐step FLA process, which is beyond the scope of this study.

The presence of a {100}‐oriented perovskite phase matching the lattice parameters of tetragonal PZT is confirmed by selected area electron diffraction (SAED) results, as shown in Figure 2e. The spaces between the reflections in the reciprocal space correspond to the (100) reflection of PZT. It should be noted that the PTO layer does not diffuse into the PZT layer during FLA, as shown in Figure S3b, Supporting Information.

Figure 2f shows the relative permittivity and dielectric loss of the PZT thin films with and without a PTO layer on top. The permittivity *ε*_r_ is 310 and 350 for the PZT thin films without and with a PTO layer on top, respectively. The corresponding dielectric loss is 3.5% and 2.7% without and with a PTO layer on top, respectively. The increase in relative permittivity is consistent with the {100} orientation, as modeled by Haun et al.^[^ ^26^, ^27^ ^]^ and reported experimentally by Coleman et al.^[^ ^28^ ^]^

The polarization and current density versus electrical field hysteresis loops with and without a PTO layer on top of the PZT thin films are shown in Figure 2g. Without PTO, a remanent polarization *P*_r_ of 4.0 μC cm^−2^, a maximum polarization *P*_max_ of 19.2 μC cm^−2^ and a coercive field *E*_c_ of 73 kV cm^−1^ are obtained. With a layer of PTO on top of the PZT thin film, values of 2.9, 21.5 μC cm^−2^ and 64 kV cm^−1^ are obtained for *P*_r_, *P*_max_ and *E*_c_, respectively. The simultaneous increase in *P*_max_ and decrease in *E*_c_ in the thin films oriented by the PTO layer as compared to the randomly oriented films without a PTO layer are consistent with literature.^[^ ^29^ ^]^ As previously discussed for the 2‐step process, the polarization versus electrical field loops are pinched and there are two distinct switching peaks at ≈8 and 180 kV cm^−1^. The relatively low remanent polarization values are attributed to the porous microstructure of the thin films (see Figure 2c,d). They are similar to the values of 5 μC cm^−2^ previously reported for 1 μm thick films with a 1‐step FLA process on thicker AF32 glass.^[^ ^11^ ^]^

### 2.3 Growing Thicker Oriented Films

**Figure 3:**
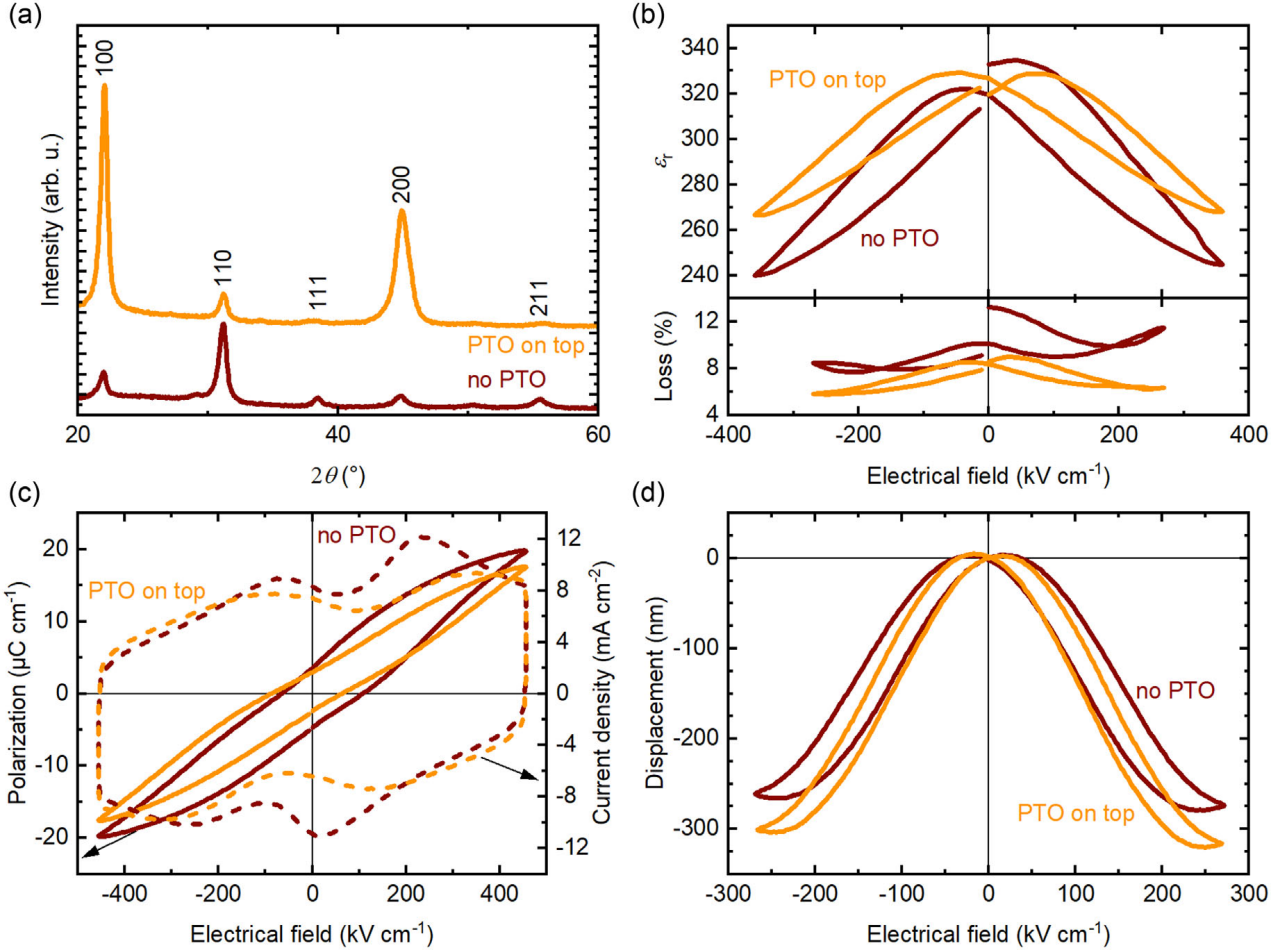
Characterization of 510 nm thick and 540 nm thick PZT films, without and with a PTO layer, respectively, on AF32 glass. a) θ−2θ XRD scans. The pseudo‐cubic *hkl* indexes correspond to the PDF card n°01‐070‐4264.^[^ ^37^ ^]^ b) Relative permittivity and dielectric loss at 1 kHz as a function of DC bias. c) Polarization (solid lines) and current density (dashed lines) versus electrical field hysteresis at 100 Hz. d) Displacement versus electrical field at 100 Hz.

To achieve a sufficiently large piezoelectric displacement, it is necessary to grow thicker PZT films. Therefore, 510 nm thick PZT films were grown on AF32 glass, both with and without a PTO layer on top. Note that with a PTO layer on top, the films effective thickness is 540 nm. To ensure the applicability of using a PTO seed layer on top of the PZT layer to crystallize thicker films by FLA, several configurations were investigated (see Section S5, Supporting Information). The best results, presented in **Figure** **3**, were obtained when each PZT layer is crystallized with a PTO layer on top.

As demonstrated for thinner films in Section 2.2, the 510 nm thick PZT thin films are randomly oriented without a PTO layer and the 540 nm thick films are strongly {100}‐textured with a PTO layer on top during the FLA. Their Lotgering factors are *f* = 0.22 and *f* = 0.82, without and with a PTO layer, respectively.

Figure 3b shows the dielectric permittivity and loss of the PZT thin films without and with a PTO layer on top. The permittivity *ε*_r_ is of 320 for the PZT thin films without a PTO layer on top and of 325 for those with a PTO layer. The corresponding dielectric loss is 10.1% and 8.4% without and with a PTO layer on top, respectively. As for thinner films (see Section 2.2), this increase in relative permittivity is consistent with the {100} orientation. The significant increase of the dielectric loss as compared to the thinner films should be noted.

The polarization versus electrical field hysteresis loops without and with a PTO layer on top of the PZT thin films are shown in Figure 3c. Without PTO, a remanent polarization *P*_r_ of 3.6 μC cm^−2^, a maximum polarization *P*_max_ of 19.8 μC cm^−2^ and a coercive field *E*_c_ of 109 kV cm^−1^ are obtained. With a layer of PTO on top of the PZT thin film, values of 3.0, 17.5 μC cm^−2^ and 67 kV cm^−1^ are obtained for *P*_r_, *P*_max_, and *E*_c_, respectively. As for thinner films (see Section 2.2), there is a decrease in *E*_c_ for the film oriented by the PTO layer. However, contrary to thinner films, *P*_max_ decreases for the film with the PTO layer. As the polarization versus electrical field loop of the PZT thin film without PTO is more rounded and the dielectric loss are larger, it is considered that the increased *P*_max_ is due to loss and that the PZT thin film with a PTO layer on top is of higher quality.

The current density versus electrical field hysteresis curves without and with a PTO layer on top of the PZT thin films are shown in Figure 3c. As for the thinner films, there are two distinct switching peaks at ≈–65 and 233 kV cm^−1^ without a PTO layer. With a PTO layer on top, the switching peaks are less pronounced, and positioned at ≈–93 and 356 kV cm^−1^. Considering the position of the second switching peak, it is possible that the 540 nm thick PZT thin film with a PTO layer on top needs higher electrical fields to reach its complete ferroelectric switching.

The piezoelectric $e_{33\text{,f}}$ coefficient is calculated from the displacement versus electrical field measurements shown in Figure 3d. As piezoelectric films show increased electromechanical response with increasing thickness due to larger contributions of domains,^[^ ^30^ ^]^ this measurement was performed on the 540 nm thick PZT thin films to obtain a significant displacement. Additionally, it allows improving the signal to noise ratio compared to $e_{33\text{,f}}$ measurements on thinner films. Without a PTO layer, a displacement of −279 nm is obtained at 91.6 V (250 kV cm^−1^) while, with PTO on top of the PZT thin film, a displacement of −320 nm is achieved at 92.6 V (249 kV cm^−1^). Considering a Young's modulus of 74.8 GPa for the AF32 glass, the piezoelectric coefficient $e_{33\text{,f}}$ is equal to 5.1 and to 5.5 C m^−2^, without and with PTO on top, respectively. These values are consistent with the $e_{33\text{,f}}$ coefficient of 5 C m^−2^ previously reported for 500 nm tick PZT thin films crystallized by FLA on 500 μm thick fused‐silica glass substrates.^[^ ^11^ ^]^ The increased $e_{33\text{,f}}$ coefficient for the PZT films with PTO on top, as compared to the films without PTO, can be ascribed to the improved quality of the films, as demonstrated by the dielectric and ferroelectric properties in Figure 2f,g.

A table summarizing the electromechanical properties of the 510 and 540 nm thick PZT films on AF32 glass is given in Section S6, Supporting Information.

### 2.4 Willow Glass: Toward Roll‐to‐Roll Processing

**Figure 4:**
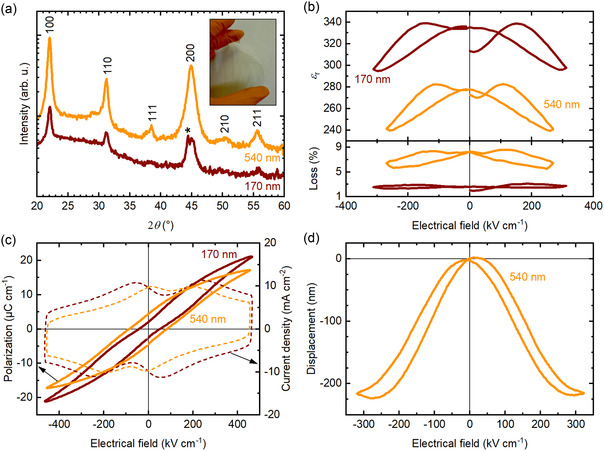
Characterization of 170 and 540 nm thick PZT thin films, with a PTO layer on top on Willow glass. a) θ−2θ XRD scans. The pseudo‐cubic *hkl* indexes correspond to the PDF card n°01‐070‐4264,^[^ ^37^ ^]^ while * denotes a signal from the sample holder. *Inset.* A picture of Willow glass. b) Relative permittivity and dielectric loss at 1 kHz as a function of DC bias. c) Polarization (solid lines) and current density (dashed lines) versus electrical field hysteresis at 100 Hz. d) Displacement versus electrical field at 100 Hz for the 540 nm thick PZT film.

To demonstrate the broader applicability of both our 2‐step FLA process and the use of a seed layer on top of the PZT layer, 170 and 540 nm thick PZT thin films have been crystallized by FLA on Willow glass. This glass is commercialized as a flexible glass, suitable for roll‐to‐roll processes. The PZT thin films on Willow glass were grown with a PTO layer on top. The FLA parameters are given in Section S3, Supporting Information and the characterization results are presented in **Figure** **4**.

From the θ−2θ XRD scans in Figure 4a, the effect of the PTO layer on top of the PZT thin films during FLA is not as pronounced on Willow glass as on AF32 glass. Yet, the Lotgering factors are *f* = 0.51 and *f* = 0.70 for the 170 nm thick and 540 nm thick PZT films, respectively. Noting the Lotgering factor of *f* = 0.87 for the 1090 nm thick PZT film shown in Figure S5, Supporting Information, there is a clear trend of improved {100}‐texture as the PZT films grow thicker on Willow glass. This is not the case on AF32 glass with *f* = 0.80, *f* = 0.82 and *f* = 0.75 for the 170 nm, 540 nm and 1090 nm thick (see Figure S5, Supporting Information) PZT films.

Figure 4b shows the dielectric permittivity and loss of the 170 nm and 540 nm thick PZT films with a PTO layer on top. With a permittivity *ε*_r_ of 335 and a dielectric loss of 2.6%, the 170 nm thick PZT thin film on Willow glass properties are close to those of a similar film on AF32 glass (see Section 2.2). However, the 540 nm thick PZT thin film has a lower permittivity on Willow glass, *ε*_r_ = 280, compared to a similar film on AF32 glass (*ε*_r_ = 327, see Section 2.3). The dielectric loss of the 540 nm thick PZT thin film on Willow glass is of 8.2%, similar to the dielectric loss of 8.4% on AF32 glass. It seems that despite the improved {100}‐texture of the 540 nm thick PZT film compared to the 170 nm thick film, it is of lower quality, possibly due to the formation of pores, leading to decreased dielectric properties. The presence of pores in the PZT thin films crystallized by FLA is show in Figure 2d and S3a, Supporting Information. It was previously reported in ref. [11].

The polarization and current density versus electrical field hysteresis loops of the 170 nm and 540 nm thick PZT films with a PTO layer on top are presented in Figure 4c. The maximum polarization *P*_max_ of 21.2 μC cm^−2^ and the coercive field *E*_c_ of 59 kV cm^−1^ of the 170 nm thick film on Willow glass are similar to the values obtained on AF32 glass (see Section 2.2). However, the remanent polarization *P*_r_ of 1.9 μC cm^−2^, is lower than that of 2.9 μC cm^−2^ obtained on AF32 glass. On the contrary, for the 540 nm thick film, the remanent polarization value of 4.5 μC cm^−2^ is larger than the value of 3.0 μC cm^−2^ obtained on AF32 glass. On Willow glass, the maximum polarization is of 17.1 μC cm^−2^, similar to the value on AF32 glass, and the coercive field is of 90 kV cm^−1^. The 170 nm thick PZT film presents two distinct switching peaks, as for the films on AF32 glass, located at 53 and 250 kV cm^−1^. The 540 nm thick film also has two switching peaks, at 17 and 204 kV cm^−1^. As for the dielectric properties, the decrease in *P*_max_ for the 540 nm thick film can be explained by a lower film quality than for the 170 nm thick PZT film on Willow glass. However, it should be noted that the polarization versus electrical field loop of the 540 nm thick film is not pinched, contrary to the one of the 170 nm thick film, leading to an improved *P*_r_.

The displacement versus electrical field measurement from which is calculated the piezoelectric $e_{33\text{,f}}$ coefficient is shown in Figure 4d. A displacement of −218 nm is obtained at 106.4 V (287 kV cm^−1^). Considering a Young's modulus of 73.6 GPa for the Willow glass, the piezoelectric coefficient $e_{33\text{,f}}$ is equal to 3.2 C m^−2^. This value is lower than the $e_{33\text{,f}}$ of 5.5 C m^−2^ obtained for the 540 nm thick PZT film with a PTO layer on top on AF32 glass. Further adjustments to the FLA process might improve the dielectric, ferroelectric and piezoelectric properties of the PZT thin films on Willow glass.

While on Willow glass thinner films display better dielectric and ferroelectric properties than thicker films (see Figure 4b,c), a piezoelectric coefficient $e_{33\text{,f}}$ of 3.2 C m^−2^ can be reached. Although fine tuning of the FLA process might be required, these results on Willow glass are very promising for the development of piezoelectric applications on flexible substrates, opening new roads for flexible and transparent electromechanical devices.

A table summarizing the electromechanical properties of the 540 nm thick PZT films on Willow glass is given in Section S6, Supporting Information.

## 3 Conclusion

In this work, we deposited ${\text{PbZr}}_{0.53}{\text{Ti}}_{0.47}O_{3}$ thin films on 100 μm thick AF32 glass substrates using the sol–gel method and crystallized them via flash lamp annealing. Through the study of FLA processes, the importance of heat management during flash lamp annealing was demonstrated, particularly for thin glass. We propose a 2‐step process which provides a power density successively suitable for the nucleation and growth of the PZT thin film. This 2‐step process prevents breaking of thin glass substrates and leads to more homogeneously crystallized thin films.

The localization of nucleation during flash lamp annealing was also investigated, and it was demonstrated that it occurs at the air–thin film interface, in contrast to nucleation occurring at the substrate‐thin film interface using conventional annealing methods. A PbTiO_3_ seed layer was deposited on top of the PZT thin film and both were crystallized simultaneously by FLA. The resulting {100} preferential orientation was confirmed by XRD and by TEM. The resulting films exhibit a piezoelectric coefficient $e_{33\text{,f}}$ of 5.5 C m^−2^, which is larger than without a PTO seed layer and larger than previously reported by FLA.^[^ ^11^ ^]^

Additionally, the results on Willow glass are promising for future applications of FLA on glass substrates fully compatible with roll‐to‐roll processes. However, complementary investigations into the formation of pores are needed to either avoid them or reduce their size, thereby improving further the quality of the PZT thin films on flexible glass substrates.

Furthermore, a wider range of flexible substrates, such as polyimide substrates, could be considered for applications in flexible electronics. These substrates, with their optical and thermal properties differing strongly from those of glass, require significant adjustments to the FLA process.

## 4 Experimental Section

4.1

4.1.1

##### Materials

The selected glass substrates were Schott AF 32 eco glass and Corning Willow glass, referred to as “AF32” glass and “Willow” glass in this article. AF32 glass was in the shape of 100 μm thick 2 in wafers. For the TEM experiments, 500 μm thick AF32 squares of 5 × 5 cm^2^ were used for ease of manipulation. Willow glass was obtained as 10 × 10 cm^2^ sheet with a thickness of 100 μm and was cleaved into smaller pieces.

${\text{Pb(Zr}}_{0.53}{\text{Ti}}_{0.47})O_{3}$ solution with a composition at the morphotropic phase boundary and a 10 mol% Pb excess was prepared following the standard 2‐methoxyethanol (99.8%, Sigma‐Aldrich) route using freeze‐dried lead (II) acetate trihydrate (99.99%, Sigma‐Aldrich), titanium (IV) isopropoxide (97%, Sigma‐Aldrich) and zirconium (IV) propoxide (70% in propanol, Sigma‐Aldrich) as starting compounds. The solution was prepared with a concentration of $C_{\text{Zr}}+C_{\text{Ti}}$ equal to 0.3 mol L^−1^ for a total volume of 100 mL. For more information, see ref. [31].

PTO solution of composition PbTiO_3_ was prepared following the same route using 1‐methoxy‐2‐propanol (99.5%, Sigma‐Aldrich) as a solvent instead of 2‐methoxyethanol. A total volume of 25 mL was prepared with a concentration of 0.1 mol L^−1^ and 30 mol% Pb excess. For more details, see ref. [32].

##### Sample Preparation

The solutions were deposited by spin‐coating for 30 s at 3000 rpm with an acceleration of 500 rpm s^−1^. The PZT and the PTO layers were dried at 130 °C for 2 min and at 135 °C for 3 min, respectively. Both were pyrolyzed at 350 °C for 2 and 3 min, respectively. The PZT amorphous films were 170 nm thick after four repetitions of the spin‐coating ‐ drying ‐ pyrolysis process. In this article, a PZT layer is understood to correspond to these four repetitions of the process, as the crystallization of PZT is always performed on such a stack. To obtain thicker films, the four repetitions of the deposition process and the crystallization process were repeated. The PTO amorphous films were 15 nm thick after one spin‐coating ‐ drying ‐ pyrolysis process. Note that the films’ thicknesses were obtained from reference PZT thin films on Pt/Si substrates crystallized by RTA.

##### Flash Lamp Annealing

Amorphous films were crystallized using a PulseForge Invent flash lamp annealer (NovaCentrix, Austin, TX, USA) equipped with a Xe lamp. The annealing parameters are the number of pulses, the repetition rate, the pulse duration, and the energy density per pulse. The last two parameters both influence the power density per pulse. The energy density was calibrated using a bolometer. An overview of the parameters is given in Section S3, Supporting Information.

##### Microstructural Characterization

Standard θ−2θ X‐ray diffraction (XRD) was performed to study the phase composition and orientation of the films using a D8 Discover diffractometer (Bruker, Billerica, MA, USA) with Cu‐Kα radiation. The patterns were recorded in the 20°–60° 2*θ* range with a step size of 0.04° and a speed of 4 s per step.

Transmission electron microscopy (TEM) analysis of the microstructure of the film was performed on a thin lamella of the sample extracted following the “lift‐out” method with a FEI (Hillsboro, OR, USA) Helios Nanolab 650 Focused Ion Beam Scanning Electron Microscope (FIB‐SEM). A JEOL (Tokyo, Japan) JEM‐F200 cold field‐emission gun (FEG) microscope operating at an acceleration voltage of 200 kV was used for dark field (DF) TEM imaging to investigate the crystalline nature of the sample. In addition, selected area electron diffraction (SAED) was performed using an aperture that selected a circular region of interest of 245 nm diameter within the film.

##### Electromechanical Characterization

Interdigitated electrodes, composed of an adhesion layer of 5 nm of Cr and a 100 nm layer of Au, were deposited on the top surface of the thin films by evaporation. The electrodes were patterned by lift‐off photolithography using a MLA 150 (Heidelberg Instruments, Heidelberg, Germany) for direct laser writing. The electrodes consisted of 50 pairs of 5 μm wide digits, separated by 3 μm, and facing each other over a length of 370 μm.

To perform electromechanical characterization, a TF Analyzer 2000 (aixACCT Systems, Aachen, Germany) was used. Relative permittivity, *ε*_r_, and dielectric loss were measured with a probing AC signal of 1 V at 1 kHz as a function of the DC voltage. Polarization versus electrical field hysteresis were measured with a triangular signal at 100 Hz.

To measure the converse piezoelectric effect,^[^ ^33^ ^]^ cantilevers measuring 25 × 3 mm^2^ were cut using a dicing saw DAD3350 (DISCO Corporation, Tokyo, Japan) equipped with a diamond blade (R07‐SDC320‐BB‐101‐75, DISCO). A picture of the cantilevers is shown in Figure S6, Supporting Information. Measurements were performed with a thin‐film sample holder unit (TFSHU, aixACCT) and a SP‐S 120 interferometer (SIOS Meßtechnik, Ilmenau, Germany). The method described by Nguyen et al.^[^ ^34^ ^]^ was used to calculate the $e_{33\text{,f}}$ coefficient, using Young's modulus values of 74.8 GPa^[^ ^35^ ^]^ and 73.6 GPa^[^ ^36^ ^]^ for the AF32 and Willow glass substrates, respectively.

## Conflict of Interest

The authors declare no conflict of interest.

## Author Contributions

**Juliette Cardoletti**: contributed to the acquisition, analysis, interpretation of data and writing of the work. **Adrian‐Marie Philippe**: contributed to the acquisition and analysis of data. **Longfei Song** and **Sebastjan Glinšek**: contributed to the acquisition of data. **Barbara Malič** and **Emmanuel Defay**: contributed to the conception of the work. **Sebastjan Glinšek**: contributed to the conception of the work and substantively revised it.

## Supporting information

Supplementary Material

## Data Availability

The data that supports the findings of the study are included in the main text and the Supplementary files. The raw data generated in this study have been deposited in the Zenodo repository under DOI: https://doi:/10.5281/zenodo.15526822.
