# Supplementary material for: Light‐Annealed Piezoelectric Films on Flexible Glass
Source: Small Sci. 2025 Jun 20;5(9):2500227. doi: 10.1002/smsc.202500227 (PMC12412497; doi:10.1002/smsc.202500227)
Supplement: Supplementary file 1 — Supplementary Material [file SMSC-5-2500227-s001.pdf]

Supplementary Information for *Light-Annealed Piezoelectric Films on Flexible Glass*

J. Cardoletti[1] *L. Song*[1] *A.-M. Philippe*[2] *S. Girod*[1] *B. Malič*[3] *E. Defay*[1,4] *S. Glinšek*[1]*

[1] Smart Materials Unit, Luxembourg Institute of Science and Technology, 41 rue du Brill, L-4422 Belvaux, Luxembourg.

[2] Advanced Analysis and Support Unit, Luxembourg Institute of Science and Technology, 41 rue du Brill, L-4422 Belvaux, Luxembourg.

[3] Electronic Ceramics Department, Jožef Stefan Institute, Jamova cesta 39, 1000 Ljubljana, Slovenia.

[4] Department of Physics and Materials Science, University of Luxembourg, 41 rue du Brill, L-4422 Belvaux, Luxembourg.

* *Corresponding author:* sebastjan.glinsek@list.lu

Contents

S1 Visual aspect of the samples . . . 2
S2 XRD comparison of 1-step and 2-step processes . . . 2
S3 Flash lamp annealing parameters . . . 3
S4 Microstructure of the films . . . 3
S5 $PbTiO_3$ seed layer configuration for thickness increase . . . 4
S6 Summary of the electromechanical properties . . . 5
S7 Characterization of 1000 nm-thick films . . . 5
S8 Cantilevers for $e_{33,f}$ measurements . . . 6

S1 Visual aspect of the samples

The visual aspect of 170 nm-thick PZT thin films on AF32 glass annealed by FLA with 1-step and 2-step processes, respectively, is shown in Figure S1. The visual inhomogeneity of the PZT thin film surface crystallized with the 1-step process is highlighted.

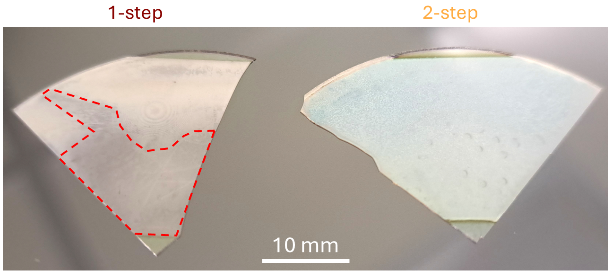

Figure S1: **Visual aspect of the samples after 1-step and 2-step FLA processes.** The area highlighted in red for the 1-step sample is visually not homogeneous compared to the rest of the sample. The regions delimited by straight lines at the bottom and at the top of the samples correspond to the clamping positions during FLA and were not exposed to the Xe lamp.

S2 XRD comparison of 1-step and 2-step processes

$\theta - 2\theta$ XRD scans around the (110) reflection of 170 nm-thick PZT films on AF32 glass annealed by FLA with 1-step and 2-step processes, respectively, are shown in Figure S2. The (110) reflection is slightly sharper for the 1-step process than for the 2-step process, indicating slightly larger crystallite size for the 1-step process as compared to the 2-step process.

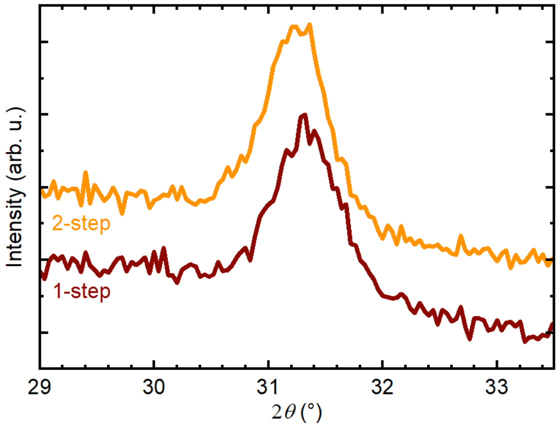

Figure S2: **$\theta - 2\theta$ XRD scans around the (110) reflection of 170 nm-thick PZT films on AF32 glass annealed by FLA with 1-step and 2-step processes, respectively.** The pseudo-cubic *hkl* indexes correspond to the PDF card n°01-070-4264 [1], while * denotes a signal from the sample holder.

S3 Flash lamp annealing parameters

The pulse duration, energy density per pulse, number of pulses and repetition rate parameters of the various flash lamp annealing (FLA) processes utilized in this work to crystallize 170 nm-thick $PbZr_{0.53}Ti_{0.47}O_3$ (PZT) films are given in **Supplementary Table S1**. The type and thickness of glass for which they are suitable are indicated in the table, along with whether a 15 nm-thick $PbTiO_3$ (PTO) buffer layer is present.

Table S1: **FLA parameters to crystallize 170 nm-thick amorphous PZT layers on AF32 and Willow glasses** of different thickness, with or without a 15 nm-thick PTO layer on top of the PZT layer.

**Suitable for**	**Process**		**Pulse duration** (μs)	**Energy density per pulse** (J cm^{-2})	**Number of pulses**	**Repetition rate** (Hz)
500 μm AF32	1-step		130	3.0	50	35
100 μm AF32	2-step	1st step	170	2.5	6	0.5
		2nd step	250	3.0	100	2.0
100 μm AF32 with PTO	2-step	1st step	200	2.5	8	0.5
		2nd step	250	3.0	100	2.0
100 μm Willow with PTO	2-step	1st step	170	2.5	6	0.5
		2nd step	250	3.0	100	2.0

S4 Microstructure of the films

To observe the microstructure of the PZT thin films crystallized by FLA and to confirm the absence of diffusion between PTO and PZT layers, transmission electron microscopy (TEM) and X-ray energy dispersive spectroscopy (EDS) line scans were performed. EDS was performed in scanning mode (STEM), using dual JEOL 100 mm^2 Silicon Drift Detectors, thus enabling the construction of elemental maps and line profile analysis. The results are shown in **Supplementary Figure S3**.

The dark-field (DF) TEM image indicates the presence of significant pores in the PZT thin films. However, the size of these pores is similar to those previously reported for PZT thin films crystallized by FLA [2]. In the DF TEM micrograph, white inclusions are visible. The contrast of these regions can arise from a difference in chemical composition or crystallographic orientation compared to the surrounding regions. Figure 2 c in the main article indicates that these inclusions have the same chemical composition as their surroundings. Therefore, the white inclusions indicate a difference in diffraction, highlighting a different crystallinity.

While the EDS line profile shows a sharp increase of both Pb-L and Ti-K signals, Zr-K profile displays a much smoother transition from the platinum top deposit toward the thin film. This is consistent with the presence of a PTO layer on top of the PZT thin film even though this first top layer might have been disturbed by the porosity growth that occurred during the annealing process. On the other side of the sample, the Si content, corresponding to the AF32 glass, starts to slowly increase before the Pb, Zr and Ti contents decrease sharply. This indicates limited diffusion of the Si into the PZT layer as previously reported for PZT thin films crystallized by FLA [2].

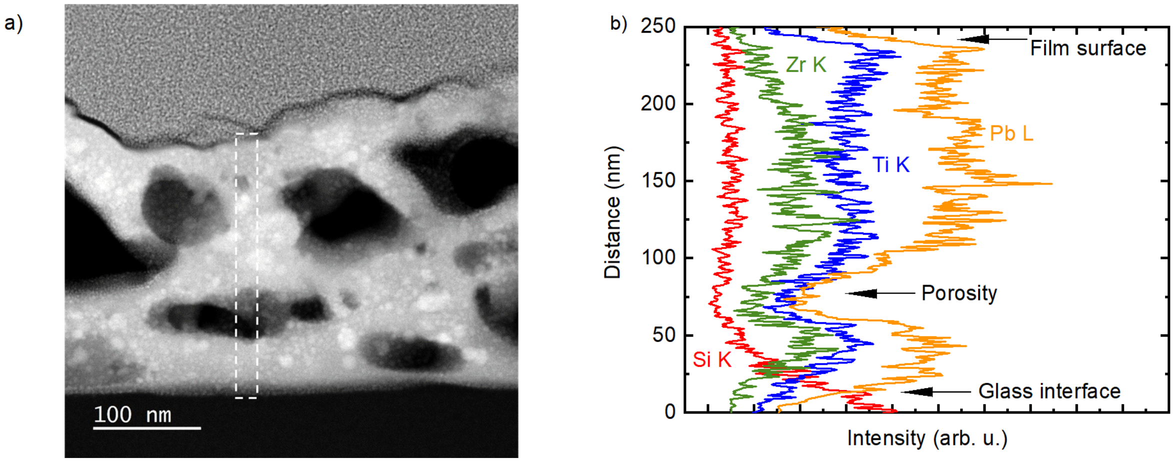

Figure S3: **a) Dark field TEM** micrograph and **b) EDS line scan of a 170 nm-thick PZT thin film with a 15 nm-thick PTO layer on top** on 500 µm-thick AF32 glass. The EDS line scan was performed across the area marked by a white dotted line in **a)**.

S5 $PbTiO_3$ seed layer configuration for thickness increase

The applicability of using a PTO layer on top of the PZT layer for thicker PZT films has been investigated and the resulting $\theta - 2\theta$ X-ray diffraction (XRD) scans are shown in **Supplementary Figure S4**. The best results are obtained when a PTO layer is applied to each PZT layer, leading to a Lotgering factor of 0.40, as opposed to 0.27 when the PTO layer is deposited only on top of the first PZT layer only. Nonetheless, the method is significantly less efficient than for 170 nm-thick PZT thin films composed of a single PZT layer for which the Lotgering factor is 0.80, as shown in the main article Section 2.2.

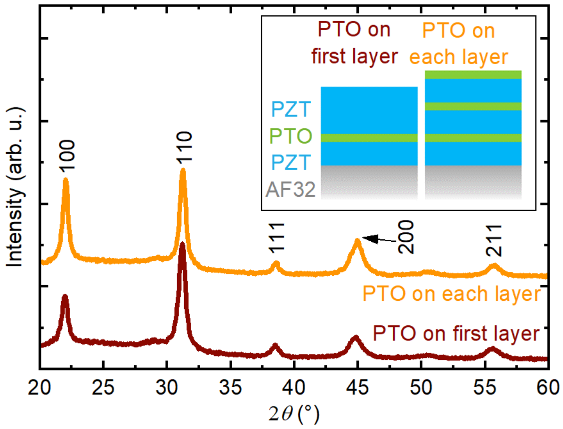

Figure S4: **θ-2θ XRD scans of PZT films with various 15 nm-thick PTO seed layer configurations**, on AF32 glass, for 540 nm-thick and 524 nm-thick PZT films with PTO on each layer and on the first layer only, respectively. The pseudo-cubic *hkl* indexes correspond to the PDF card n°01-070-4264 [1]. *Inset.* Schematic representation of the various configurations tested for the PTO layer(s), not to scale.

S6 Summary of the electromechanical properties

The electromechanical properties of 510 nm-thick PZT thin films on 100 μm-thick AF32 and Willow glass, without and with a PTO layer on top, are given in **Supplementary Table S2**.

Table S2: **Summary of the electromechanical properties of 510 nm-thick PZT thin films** on 100 μm-thick AF32 glass, without and with a PTO layer on top, as well as on Willow glass, with a PTO layer on top. The PZT films with a PTO layer on top are 540 nm-thick.

**Property**	**AF32 glass**		**Willow glass**
	Without PTO	With PTO on top	With PTO on top
ϵ_r at 1 kHz	320	325	280
Dielectric loss(%) at 1 kHz	10.1	8.4	8.2
P_r (μC cm^{-2}) at 100 Hz	3.6	3.0	4.5
P_{max} (μC cm^{-2}) at 100 Hz	19.8	17.5	17.1
E_c (kV cm^{-1}) at 100 Hz	109	67	90
$e_{33,f}$ (C m^{-2}) at 100 Hz	5.1	5.5	3.2

S7 Characterization of 1000 nm-thick films

To confirm the applicability of our method to thicker films, 1090 nm-thick PZT films with a PTO layer on top were grown on both AF32 glass and Willow glass. The $\theta - 2\theta$ XRD scans of these films are shown in **Supplementary Figure S5**.

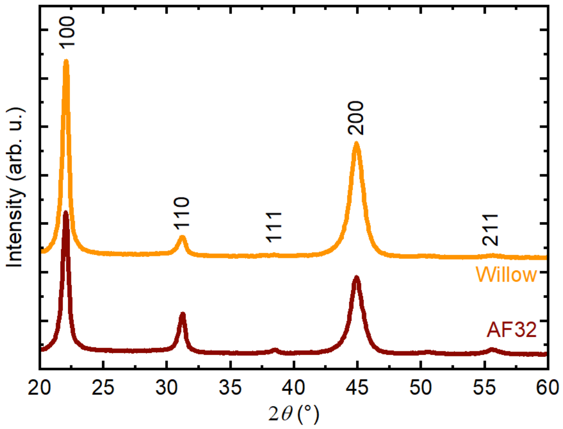

Figure S5: **θ-2θ XRD scan of a 1090 nm-thick PZT film**, with a PTO layer on top, on both AF32 and Willow glass. The pseudo-cubic *hkl* indexes correspond to the PDF card n°01-070-4264 [1].

As shown for thinner films in the main article Section 2.2 and Section 2.4, the 1090 nm-thick PZT thin films with a PTO layer on top during the FLA are strongly {100}-textured. Their Lotgering factors are f = 0.75 and f = 0.87 on AF32 glass and Willow glass, respectively.

S8 Cantilevers for $e_{33,\mathrm{f}}$ measurements

A picture of a 25×3 mm^2 cantilever used to measure the converse piezoelectric effect and to calculate the $e_{33,\mathrm{f}}$ coefficient is shown in Figure S6.

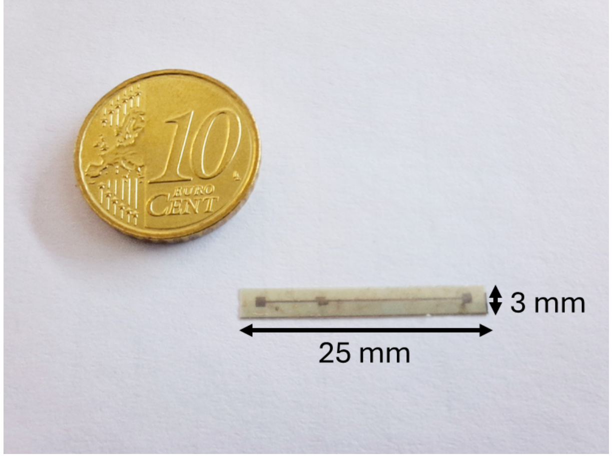

Figure S6: **A picture of a 25×3 mm^2 cantilever to measure the converse piezoelectric effect**.

References

[1] ICDD database PDF4+ v.19, 2019.

[2] L. Song, J. Cardoletti, A. Blázquez Martínez, A. Benčan, B. Kmet, S. Girod, E. Defay, and S. Glinšek. Crystallization of piezoceramic films on glass via flash lamp annealing. *Nature Communications*, 15(1):1890, Feb. 2024.
